# Study on a Plasmonic Tilted Fiber Grating-Based Biosensor for Calmodulin Detection

**DOI:** 10.3390/bios11060195

**Published:** 2021-06-14

**Authors:** Xiaoyong Chen, Jie Jiang, Nan Zhang, Wenwei Lin, Pin Xu, Jinghua Sun

**Affiliations:** 1 School of Electrical Engineering and Intelligentization, Dongguan University of Technology, Dongguan 523808, China; sunjh@dgut.edu.cn; 2 Department of Physics, Shantou University, Shantou 515063, China; 18jjiang@stu.edu.cn (J.J.); 18nzhang4@stu.edu.cn (N.Z.); wwlin@stu.edu.cn (W.L.); 19pxu@stu.edu.cn (P.X.)

**Keywords:** fiber-optic biosensor, tilted fiber Bragg grating, surface plasmonic resonance, calmodulin, limit of detection

## Abstract

Tilted fiber Bragg grating, which has the advantages of both fiber Bragg grating and long-period fiber grating, has been widely studied for sensing in many fields, especially in the field of biochemistry. Calmodulin, which has a wide distribution in eukaryotes, can regulate several enzymes such as adenylate cyclase and guanylate cyclase and mediates several cellular processes such as cell proliferation and cyclic nucleotide metabolism. The abnormal levels of calmodulin in the body will result in serious effects from metabolism to nerve growth and memory. Therefore, it is important to measure the calmodulin concentration in the body. In this work, we propose and experimentally demonstrate a plasmonic tilted fiber Bragg grating-based biosensor for calmodulin detection. The biosensor was made using an 18° tilted fiber Bragg grating with a 50 nm-thick gold nanofilm coating the surface of the fiber, and transient receptor potential channels were bonded onto the surface of the gold nanofilm to serve as bio-detectors for calmodulin detection. Experimental results showed that the limit of detection using our biosensor was 0.44 nM. Furthermore, we also demonstrated that the interaction between calmodulin and transient receptor potential channels was quite weak without calcium in the solution, which agrees with the biology. Our proposed biosensor has a simple structure, is easy to manufacture, and is of small size, making it a good choice for real-time, label-free, and microliter-volume biomolecule detection.

## 1. Introduction

Because of many desirable advantages, such as small size, remote control, immunity from electromagnetic fields, and biocompatibility, fiber-optic sensors have been increasingly considered for real-time and label-free biochemical sensing in recent years [1,2]. Among all fiber-optic sensors, tilted fiber Bragg grating (TFBG), in which the refractive index modulation planes are angled by a few degrees relative to the propagation axis, has attracted great attention because it can measure small changes in the surrounding refractive index near the surface of the fiber while simultaneously measuring the temperature for calibrating the temperature-induced cross-sensitivity [3,4,5]. Moreover, TFBG is usually inscribed into the fiber core by using phase-mask or femtosecond laser techniques, which will not introduce any breaks in the structure of the fiber, making it more stable compared to other fiber sensors, such as D-shaped [6,7] and tapered fiber sensors [8,9,10]. Furthermore, owing to the tilt grating-induced break in the cylindrical symmetry of the fiber, some of the power propagating in the core of the fiber can be coupled to the fiber cladding, exciting hundreds of cladding modes traveling backward in the cladding [11]. The excited cladding modes can be observed as a high-density comb of narrowband spectral resonances (with a Q-factor of 10^4^ [12]) in the transmission spectrum, covering a wavelength band of tens of nanometers. The break in cylindrical symmetry also results in a strong polarization selectivity of the excited cladding modes, enabling the excitation of surface plasmon resonances (SPR) in a metallic nanofilm that coats the surface of the fiber. TFBG coated with a metallic nanofilm, also called plasmonic TFBG, has both the advantages of TFBG and SPR [13]. Compared to the conventional TFBG without a metallic nanofilm, the plasmonic TFBG has a great advantage in that the electromagnetic energy on the metallic surface is stronger, resulting in it being more sensitive to the surrounding refractive index [14]. In the past few years, TFBG has been widely studied for biochemical sensing, involving a non-enzymatic D-glucose biosensor (limit of detection (LOD) 10^−8^ M, detection range 10^−8^–10^−2^ M) [15], a breast cancer biomarker biosensor (LOD 10^−12^ g/mL) [16], a thrombin molecule biosensor (LOD 2.5 nM, 2.5–40 nM) [17], a circulating tumor cell detector (LOD 10 cancer cells/mL) [18], a cytokeratin biosensor (LOD 14 pM) [19], a mercury Ions detector (LOD 3.073 pM, dynamic range 10^−11^–10^−3^ M) [20], a glucose detector (LOD 295 pM, dynamic range 1 nM–10 mM) [21], a small biomolecule biosensor (LOD 1 nM) [22], and a hydrogen sensor (LOD 180 ppm) [23]. In summary, the LOD of TFBG for biochemical sensing can reach the nanomole level or even the picomole level. Therefore, TFBG-based sensors can achieve the LOD required for biomedical and biochemical reactions [24,25,26], enabling single-point biomedical sensing in hard-to-reach spaces, such as in vivo, to be possible.

Calcium ions (Ca^2+^) affect almost all physiological activities, and calcium signaling is common for signaling either between cells or within cells [27]. Many proteins, such as troponin C, parvalbumin, calmodulin (CaM), and myosin light chains, can bind calcium, but calmodulin is the most common calcium-modulated protein, as it has a wide distribution in eukaryotes and mediates several cellular processes, including cell proliferation, gene expression, cyclic nucleotide metabolism, ion channel activities, protein phosphorylation and dephosphorylation, cell Ca^2+^ metabolism, and others [28,29,30]. In other words, the effects caused by calmodulin range from inflammation and metabolism to nerve growth and memory [31,32]. As calmodulin plays an important role in cell cycle regulation, either directly by regulating the function of cell cycle proteins or indirectly by activating calmodulin-dependent kinases and phosphatases crucial for cell cycle regulation [33], it is significant to monitor the calmodulin levels in the body.

In this work, we demonstrate a plasmonic TFBG biosensor, which was made by a 50 nm thick gold nanofilm coating the fiber surface, followed by bonding transient receptor potential (TRP) channels onto the surface of the gold nanofilm for acting as bio-receptors [34], for calmodulin detection. To simplify the implementation process, the sensor was designed as a reflective probe by depositing a gold mirror downstream of the TFBG. The LOD of the proposed biosensor was studied in this work, together with the interaction between CaM and TRP in solution with and without Ca^2+^.

## 2. Materials and Methods

### 2.1. Materials

All biomolecule-related materials, including CaM (1 mM), TRP (1 mM), and buffer solutions were provided by Yukun Cui, and they were purchased from Shanghai Macklin Biochemical Co., Ltd (Shanghai, China). All chemicals, including 11-mercaptoundecanoic acid, 1-ethyl-3-(3-dimethylaminopropyl) carbodiimide hydrochloride (EDC), and N-hydroxysuccinimide (NHS) were purchased from Wuhan Boster Biological Engineering Co., Ltd., Wuhan, China. 

### 2.2. Fabrication of the TFBG-Based Biosensor

**Figure 1:**
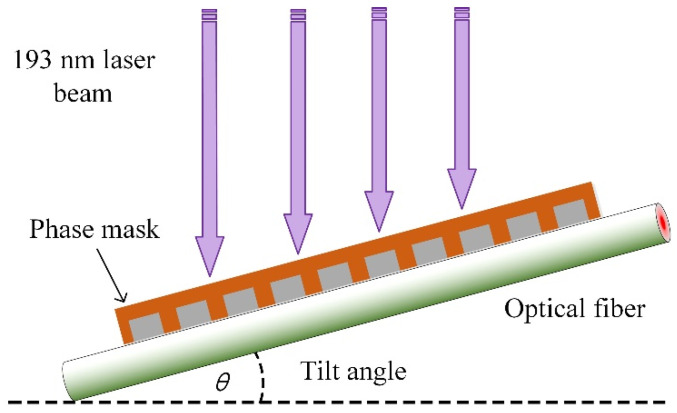
Detail of the phase-mask technique for TFBG fabrication.

The TFBG used in the work was fabricated using the phase-mask technique (±1 diffraction order) [35], which can be seen in Figure 1. To increase the photosensitivity of the fiber core, a germanium-doped silica fiber, instead of a commercial single-mode fiber, was used for tilted grating inscription. An excimer laser with a wavelength of 193 nm, 3 mJ of power per pulse, and a frequency of 200 Hz, was used as the light source. The laser beam scanned the phase-mask during the inscription procedure to improve the quality of tilt grating. The TFBG transmission spectrum can be expressed as Equations (1) and (2) [4]:

(1) $$\lambda_{i}=\left(N_{eff}^{core}\left(\lambda_{i}\right)+N_{eff}^{i}\left(\lambda_{i}\right)\right)\cdot \Lambda /\cos\theta$$

(2) $$R_{i}=\tanh^{2}\left(\kappa_{i}L\right)$$

where $\lambda_{i}$ and $R_{i}$ represent the resonant wavelength and the strength of the resonance, respectively. $N_{eff}^{core}\left(\lambda_{i}\right)$ and $N_{eff}^{i}\left(\lambda_{i}\right)$ are the effective indices of the guided-mode in the core and the exciting cladding mode i at the resonant wavelength $\lambda_{i}$, respectively; Λ is the grating period, θ is the tilt angle; $\kappa_{i}$ is the coupling coefficient, and L is the grating length. In this work, a TFBG with a tilt angle of 18° was selected for the experiments.

To excite SPR, a 50 nm-thick gold nanofilm was deposited on the surface of the fabricated TFBG (as shown in Figure 1) through the radio-frequency magnetron sputtered method [36]. As the fiber is a cylindrical structure, to obtain a more uniform film, the fiber was continually rotated about the fiber axis at a speed of 0.5 rad/s during the deposition process. Although we can determine the thickness of the gold deposited on the fiber surface by controlling the working time of the radio-frequency magnetron sputtered machine, we could not, one hundred percent, ensure that the gold thickness was 50 nm each time. What we could guarantee was that the gold thickness was approximately 50 nm according to our previous experience, and the SPR could be strongly excited. After that, a gold mirror was deposited, using the same method, downstream of the TFBG, making the plasmonic TFBG work as a reflection probe. The SPR excited by the TFBG can be understood when the propagation constants of the cladding modes are equal to those of the surface plasmon polaritons (SPPs), power coupling between the cladding modes and SPPs can occur, and this phenomenon is observed as a consequent reduction in the TFBG spectrum. Small changes near the metallic surface could be measured by monitoring the power changes of the cladding modes within the SPR absorption area. 

**Figure 2:**
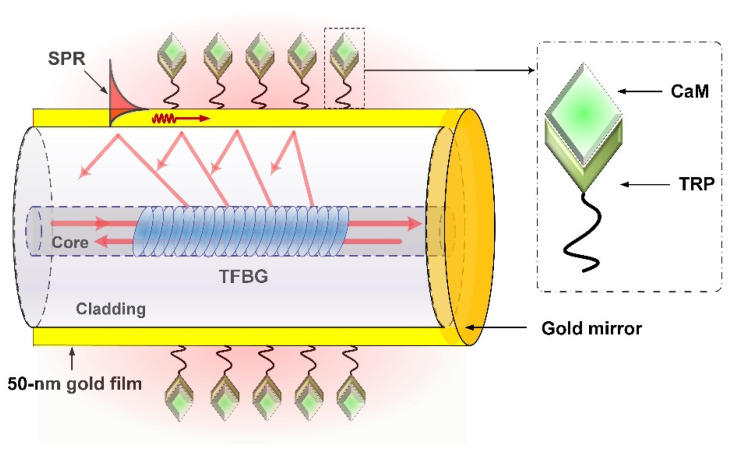
Detail of the proposed sensor.

In order to make a specific detection, surface functionalization for the plasmonic TFBG should be carried out. As the TRP can specifically interact with CaM, we used TRP as a bio-receptor and bonded it onto the metallic surface, as shown in Figure 2. The surface functionalization included several steps as follows:

(1) The plasmonic TFBG was rinsed with ethanol and with Milli-Q water to remove unwanted contaminants on the metallic surface, and then was immersed in the 11-mercaptoundecanoic acid solution (~10 µM) for 2 h to allow the self-assembly of a monolayer of mercapto compounds on the metallic surface;
(2) The plasmonic TFBG was again rinsed with ethanol and with Milli-Q water for removing the nonadherent 11-mercaptoundecanoic acid, and then was immersed in a mixed solution that contained 1.5 mL of EDC (50 mM) and 0.5 mL of NHS (50 mM) for 30 min to activate the carboxyl groups on the self-assembled monolayer;
(3) The sensor was rinsed with the reaction buffer consisting of 50 mM Tris-HCl, 100 mM NaCl, 1 mM DTT, and 1 mM CaCl_2_ at PH 7.5, for removing the nonadherent EDC and NHS;
(4) The sensor was immersed in the TRP solution (10 µM) for 1 h to bind the TRP to the metallic surface. After that, the biosensor was ready for calmodulin detection.

### 2.3. Experimental Design

**Figure 3:**
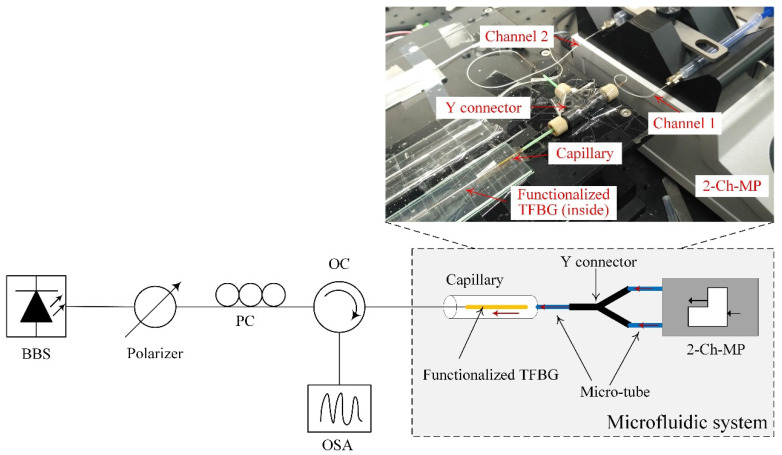
Block diagram of experimental setup. BBS: broadband source; PC: polarization controller; OC: optical circulator; OSA: optical spectrum analyzer; 2-Ch-MP: 2-channel-micro-pump.

Figure 3 shows the block diagram of the experimental setup used in this work. A broadband source (1460–1560 nm) followed by an in-line fiber polarizer was the light source. A manual paddle fiber polarization controller was used to control the polarization state of the light launched into the TFBG. An optical circulator was applied to connect the TFBG to an optical spectrum analyzer, which was used to monitor and record the reflected spectra from the TFBG. The inset in Figure 2 shows the practical microfluidic system used in the experiment. The inner diameter of the micro-tube and the capillary (with a length of 5 cm) was 300 µM. Because of the custom-designed microfluidic system, the sample solution required for the experiment was only 20 µL. Additionally, a two-channel-pump was used to inject the buffer and sample solutions independently, thus making the implementation easier. 

## 3. Results and Discussion

**Figure 4:**
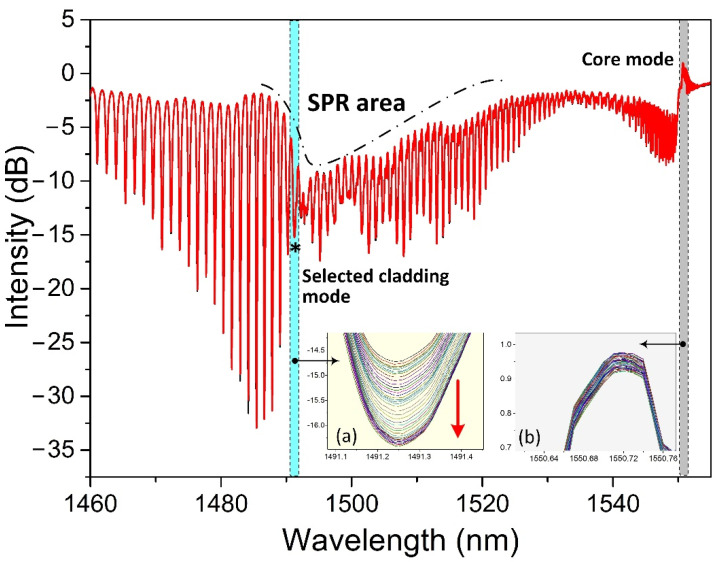
A measured spectrum of the biosensor. Insets (**a**,**b**) are, respectively, the responses of the selected cladding mode and core mode when the biosensor was used for measuring CaM at a concentration of 1 μM.

Figure 4 shows a measured spectrum of the proposed biosensor when it was immersed in a CaM solution. Based on this spectrum, we focused mainly on two spectral regions during the measuring procedure: one was the SPR absorption area, and the other was the core mode. The cladding modes within the SPR absorption area were sensitive to perturbations near the metallic surface, especially the cladding mode resonances adjacent to the center of the SPR absorption area. Therefore, we selected the first cladding mode resonance, indicated with a black star “*” on the left side next to the SPR center for monitoring the detection procedure. We should point out here that the TFBG amplitude spectrum to the right of the SPR area appeared quite noisy. This power fluctuation of some cladding modes was caused during the TFBG fabrication. However, the experimental tests in sensitivity and stability have demonstrated that it could provide performances as other TFBGs. Furthermore, as we select the first cladding mode to the left of the SPR center for demodulation, this “noise” to the right of the SPR area will not affect the demodulated result in this work. The core was used to calibrate the temperature-induced cross-sensitivity and light source-induced intensity fluctuation in this work. Insets (a) and (b) show, respectively, the responses of the selected cladding mode resonance and the core mode when the biosensor was immersed in a 1 μM CaM solution for 30 min. Note that the optical spectrum analyzer automatically recorded the spectrum every 30 s. The intensity of the selected cladding mode decreased with time and became stable after a certain time. The intensity fluctuation, based on the core mode shown in inset (b), was only ~0.05 dB, far smaller than the intensity change in the selected cladding mode (~1.7 dB). Therefore, we could ignore the power fluctuation-induced effect in the experiment. Furthermore, it was not necessary to calibrate the temperature-induced cross-sensitivity, as almost no wavelength shift occurred according to the core mode. 

**Figure 5:**
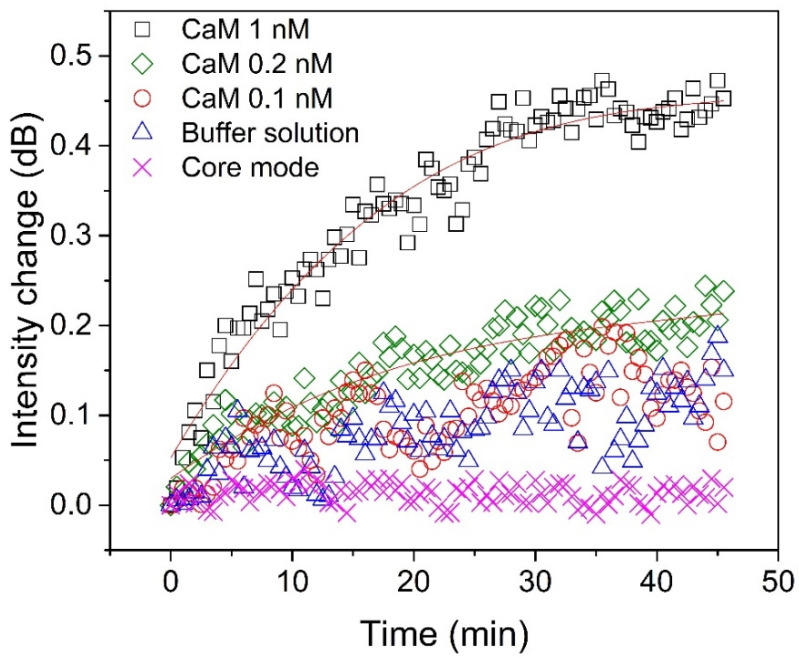
Detection of calmodulin at concentrations of 1 nM (black “□”), 0.2 nM (green “◇”), and 0.1 nM (red “○”) compared with buffer solution without calmodulin (blue “∆”). The intensity changes of the core mode (pink “X”), at 1540 nm, as it varied with time is also shown.

**Figure 6:**
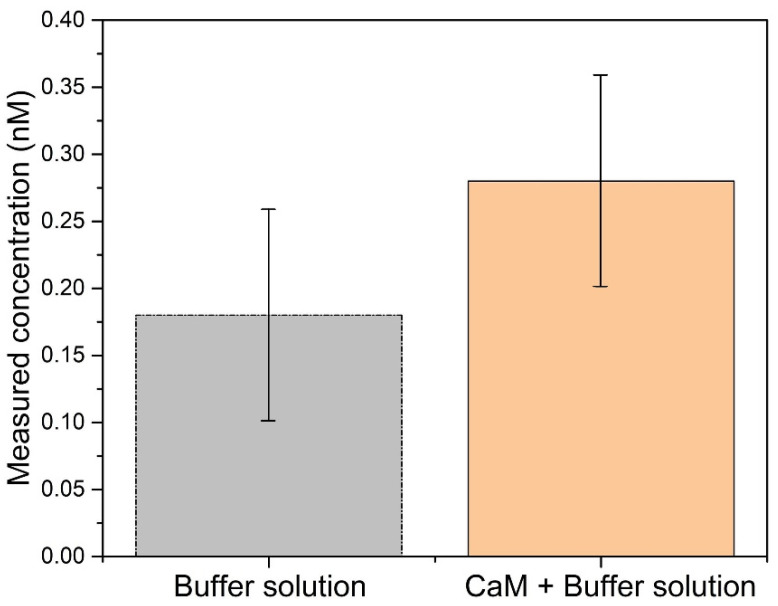
Measured concentrations using the TFBG functionalized with TRP immersed in buffer solution, in the presence or in the absence of CaM.

To evaluate the LOD of the proposed biosensor, several experiments were carried out for measuring CaM at different concentrations (Figure 5). As can be seen, the intensity change of the selected cladding mode was clear when the CaM concentration was 1 nM. The change in intensity increases quickly during the first 30 min, and then rises slowly and becomes almost saturated after 40 min, which means that the interaction between CaM and TRP channels reaches equilibrium. When the CaM concentration was 0.2 nM, the analytical signal can be distinguished from the background signal (buffer solution). Although the change in intensity in the selected cladding mode can also be observed when the CaM concentration is 0.1 nM (red hollow circles), it is difficult to distinguish it from that caused by the buffer solution (blue hollow triangles). To precisely calculate the actual LOD of our biosensor, we repeated the experiments ten times, and the results are shown in Figure 6. The LOD can be computed according to the following formulas [37]:

(3) $$LOD=LOB+1.645\cdot SD_{L}$$

(4) $$LOB=mean_{blank}+1.645\cdot SD_{blank}$$

where LOB (limit of blank) is defined as the highest apparent analyte concentration expected to be found when replicates of a sample containing no analyte are tested. $SD_{L}$ and $SD_{blank}$ represent the standard deviations of the blank and the lowest concentration samples in a ten-time measurement, respectively. $mean_{blank}$ is the mean result of the blank sample (buffer solution). According to Figure 6, it could be calculated that the LOD of our biosensor was 0.44 nM.

In addition, the power fluctuation from the core mode, indicated by the pink “X”, was only ~0.03 dB during the experiment; thus, we could ignore its induced effect. It needs to be highlighted that the buffer solutions used for diluting the CaM concentration or for stability testing contained 1 mM Ca^2+^, as it was required for the interaction between CaM and TRP. 

**Figure 7:**
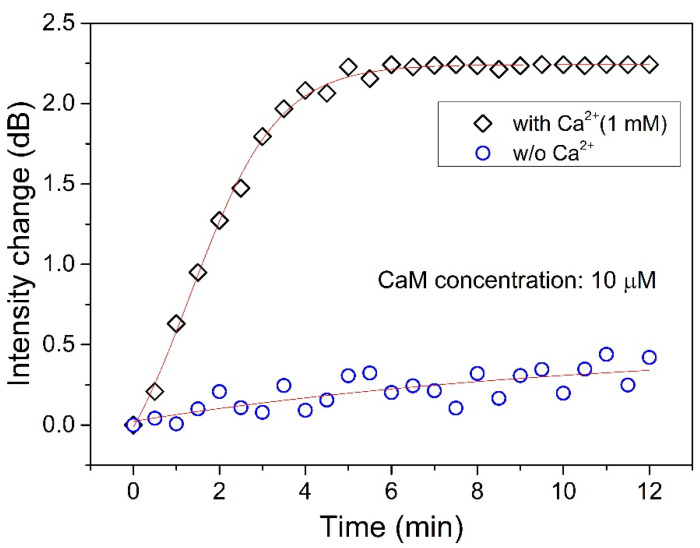
Interaction monitoring between CaM (10 μM) and TRP channels in solutions with a Ca^2+^ concentration of 1 mM (labeled “◇”) and without Ca^2+^ (labeled “○”).

Finally, we also tested the interaction between CaM and TRP channels in solution with and without Ca^2+^ (Figure 7). Note that the Ca^2+^ concentration used in the experiment was 1 mM, as the Ca^2+^ concentration in the body is slightly higher than this value. We can see that even though the CaM concentration is 10 μM, the interaction between CaM and TRP was very weak (as the blue circles in Figure 5 show) if there is no Ca^2+^ in the solution. On the other hand, the interaction was very strong when the Ca^2+^ was present in the solution, as the black squares show. The change in intensity increased sharply in the first 4 min, and it reached saturation after 5 min, at which point the interaction reached equilibrium.

Because of the custom-design microfluidic system, bio-sample solutions can be automatedly injected into the capillary, which acts as a bio-sample cell in the system for measurements. It was easy to monitor in real-time the interaction between the targeted biomolecule and the bio-receptor on the sensor surface. Therefore, our proposed plasmonic TFBG, together with the microfluidic system, can not only be used for biomolecule detection but also for interaction monitoring between biomolecules. 

## 4. Conclusions

A plasmonic TFBG-based biosensor, in which the TRP channels were bonded onto the metallic surface for acting as bio-receptors, was demonstrated for calmodulin detection in this work. Experimental results showed that the LOD of our biosensor was 0.44 nM, and the operating time was 40 min. Additionally, our proposed sensor combined with a microfluidic system can also be used for monitoring the interaction between CaM and TRP channels in real-time. Our proposed plasmonic TFBG sensor, together with the custom-designed microfluidic system, offers possibilities for practical biomolecule detection and biomolecule interaction monitoring in the future, owing to its characteristics of fast response, easy to manufacture, automatic control, and a microliter volume requirement for the bio-sample solution. 

## Data Availability

All data are contained within the article.

## References

[1] Dai M., Chen Z., Zhao Y., Aruna Gandhi M.S., Li Q., Fu H. (2020). State-of-the-Art Optical Microfiber Coupler Sensors for Physical and Biochemical Sensing Applications. Biosensors.

[2] Ma P., Hu N., Ruan J., Song H., Chen X. (2020). In-Situ Measurement of Ammonium in Wastewater using a Tilted Fiber Grating Sensor. J. Lightwave Technol..

[3] Jiang B., Zhou K., Wang C., Sun Q., Yin G., Tai Z., Wilson K., Zhao J., Lin Z. (2017). Label-free glucose biosensor based on enzymatic graphene oxide-functionalized tilted fiber grating. Sens. Actuators B Chem..

[4] Albert J., Shao L.Y., Caucheteur C. (2013). Tilted fiber Bragg grating sensors. Laser Photon. Rev..

[5] Chen X., Xu J., Zhang X., Guo T., Guan B.O. (2017). Wide Range Refractive Index Measurement Using a Multi-Angle Tilted Fiber Bragg Grating. IEEE Photon. Technol. Lett..

[6] Jiang B., Bai Z., Wang C., Zhao Y., Zhao J., Zhang L., Zhou K. (2018). In-Line Mach-Zehnder Interferometer with D-Shaped Fiber Grating for Temperature-Discriminated Directional Curvature Measurement. J. Lightwave Technol..

[7] Dong Y., Xiao S., Wu B., Xiao H., Jian S. (2019). Refractive Index and Temperature Sensor Based on D-Shaped Fiber Combined with a Fiber Bragg Grating. IEEE Sens. J..

[8] Cano Perez J.L., Gutiérrez-Gutiérrez J., Perezcampos Mayoral C., Pérez-Campos E.L., del Socorro Pina Canseco M., Tepech Carrillo L., Mayoral L.P., Vargas Treviño M., Apreza E.L., Rojas Laguna R. (2021). Fiber Optic Sensors: A Review for Glucose Measurement. Biosensors.

[9] Zhao Y., Cai L., Hu H. (2015). Fiber-Optic Refractive Index Sensor Based on Multi-Tapered SMS Fiber Structure. IEEE Sens. J..

[10] Ping L. (2009). Tapered fiber Mach-Zehnder interferometer for simultaneous measurement of refractive index and temperature. Appl. Phys. Lett..

[11] Dong X., Zhang H., Liu B., Miao Y. (2011). Tilted fiber Bragg gratings: Principle and sensing applications. Photon. Sens..

[12] Guo T., Liu F., Guan B.-O., Albert J. (2016). Tilted fiber grating mechanical and biochemical sensors. Opt. Laser Technol..

[13] Albert J., Lepinay S., Caucheteur C., DeRosa M.C. (2013). High resolution grating-assisted surface plasmon resonance fiber optic aptasensor. Methods.

[14] Caucheteur C., Guo T., Albert J. (2015). Review of plasmonic fiber optic biochemical sensors: Improving the limit of detection. Anal. Bioanal. Chem..

[15] Lobry M., Lahem D., Loyez M., Debliquy M., Chah K., David M., Caucheteur C. (2019). Non-enzymatic D-glucose plasmonic optical fiber grating biosensor. Biosens. Bioelectron..

[16] Lobry M., Loyez M., Hassan E.M., Chah K., DeRosa M.C., Goormaghtigh E., Wattiez R., Caucheteur C. (2020). Multimodal plasmonic optical fiber grating aptasensor. Opt. Express.

[17] Sypabekova M., Korganbayev S., González-Vila Á., Caucheteur C., Shaimerdenova M., Ayupova T., Bekmurzayeva A., Vangelista L., Tosi D. (2019). Functionalized etched tilted fiber Bragg grating aptasensor for label-free protein detection. Biosens. Bioelectron..

[18] Loyez M., Hassan E.M., Lobry M., Liu F., Caucheteur C., Wattiez R., DeRosa M.C., Willmore W.G., Albert J. (2020). Rapid Detection of Circulating Breast Cancer Cells Using a Multiresonant Optical Fiber Aptasensor with Plasmonic Amplification. ACS Sens..

[19] Loyez M., Albert J., Caucheteur C., Wattiez R. (2018). Cytokeratins biosensing using tilted fiber gratings. Biosensors.

[20] Duan Y., Wang F., Zhang X., Liu Q., Lu M., Ji W., Zhang Y., Jing Z., Peng W. (2020). TFBG-SPR DNA-Biosensor for Renewable Ultra-Trace Detection of Mercury Ions. J. Lightwave Technol..

[21] Wang F., Lu M., Yuan H., Zhang Y., Ji W., Sun C., Peng W. (2021). pM Level and Large Dynamic Range Glucose Detection Based on a Sandwich Type Plasmonic Fiber Sensor. J. Lightwave Technol..

[22] Chen X., Nan Y., Ma X., Liu H., Liu W., Shi L., Guo T. (2019). In-Situ Detection of Small Biomolecule Interactions Using a Plasmonic Tilted Fiber Grating Sensor. J. Lightwave Technol..

[23] Cai S., González-Vila L., Zhang X., Guo T., Caucheteur C. (2019). Palladium-coated plasmonic optical fiber gratings for hydrogen detection. Opt. Lett..

[24] Zhang Y., Wang F., Qian S., Liu Z., Wang Q., Gu Y., Wu Z., Jing Z., Sun C., Peng W. (2017). A Novel Fiber Optic Surface Plasmon Resonance Biosensors with Special Boronic Acid Derivative to Detect Glycoprotein. Sensors.

[25] Lao J., Sun P., Liu F., Zhang X., Zhao C., Mai W., Guo T., Xiao G., Albert J. (2018). In situ plasmonic optical fiber detection of the state of charge of supercapacitors for renewable energy storage. Light Sci. Appl..

[26] Ribaut C., Loyez M., Larrieu J.-C., Chevineau S., Lambert P., Remmelink M., Ruddy W., Caucheteur C. (2016). Cancer biomarker sensing using packaged plasmonic optical fiber gratings: Towards in vivo diagnosis. Biosens. Bioelectron..

[27] Munk M., Alcalde J., Lorentzen L., Villalobo A., Berchtold M.W., Panina S. (2020). The impact of calmodulin on the cell cycle analyzed in a novel human cellular genetic system. Cell Calcium.

[28] Sharma R.K., Parameswaran S. (2018). Calmodulin-binding proteins: A journey of 40 years. Cell Calcium.

[29] Zhang M., Abrams C., Wang L., Gizzi A., He L., Lin R., Chen Y., Loll P.J., Pascal J.M., Zhang J.F. (2012). Structural Basis for Calmodulin as a Dynamic Calcium Sensor. Structure.

[30] Mehta D., Negi S., Ganesh R. (2020). Molecular dynamics simulations to study the interaction between carbon nanotube and calmodulin protein. Mater. Today Proc..

[31] Clapham D.E. (2007). Calcium signaling. Cell.

[32] Hudmon A., Schulman H., Lennarz W.J., Lane M.D. (2013). Calcium/Calmodulin-Dependent Protein Kinase II. Encyclopedia of Biological Chemistry.

[33] Hurley R.L., Anderson K.A., Franzone J.M., Kemp B.E., Means A.R., Witters L.A. (2005). The Ca^2+^/Calmodulin-dependent Protein Kinase Kinases Are AMP-activated Protein Kinase Kinases. J. Biol. Chem..

[34] Sun Z., Zheng Y., Liu W. (2018). Identification and characterization of a novel calmodulin binding site in Drosophila TRP C-terminus. Biochem. Biophys. Res. Commun..

[35] Caucheteur C., Guo T., Albert J. (2017). Polarization-Assisted Fiber Bragg Grating Sensors: Tutorial and Review. J. Lightwave Technol..

[36] Chen X., Du F., Guo T., Lao J., Zhang X., Zhang Z., Liu F., Li J., Chen C., Guan B.O. (2017). Liquid Crystal-Embedded Tilted Fiber Grating Electric Field Intensity Sensor. J. Lightwave Technol..

[37] Armbruster D.A., Pry T. (2008). Limit of blank, limit of detection and limit of quantitation. Clin. Biochem. Rev..

